# Augmented Reality in Ultrasound-Guided Regional Anaesthesia: An Exploratory Study on Models With Potential Implications for Training

**DOI:** 10.7759/cureus.42346

**Published:** 2023-07-24

**Authors:** Sean P Shevlin, Lloyd Turbitt, David Burckett-St.Laurent, Alan J Macfarlane, Simeon West, James S Bowness

**Affiliations:** 1 Anaesthesia, Belfast Health and Social Care Trust, Belfast, GBR; 2 Anaesthesia, Royal Gwent Hospital, Newport, Truro, GBR; 3 Anaesthesia, Glasgow Royal Infirmary, Glasgow, GBR; 4 Anaesthesia, University College London Hospital, London, GBR; 5 Anaesthesia, Aneurin Bevan University Health Board, Newport, GBR

**Keywords:** medical education, artificial intelligence and education, augmented reality, ultrasound guided regional anaesthesia, regional anaesthesia

## Abstract

Introduction

Needle tip visualisation is a key skill required for the safe practice of ultrasound-guided regional anaesthesia (UGRA). This exploratory study assesses the utility of a novel augmented reality device, NeedleTrainer™, to differentiate between anaesthetists with varying levels of UGRA experience in a simulated environment.

Methods

Four groups of five participants were recruited (n = 20): novice, early career, experienced anaesthetists, and UGRA experts. Each participant performed three simulated UGRA blocks using NeedleTrainer™ on healthy volunteers (n = 60). The primary aim was to determine whether there was a difference in needle tip visibility, as calculated by the device, between groups of anaesthetists with differing levels of UGRA experience. Secondary aims included the assessment of simulated block conduct by an expert assessor and subjective participant self-assessment.

Results

The percentage of time the simulated needle tip was maintained in view was higher in the UGRA expert group (57.1%) versus the other three groups (novice 41.8%, early career 44.5%, and experienced anaesthetists 43.6%), but did not reach statistical significance (p = 0.05). An expert assessor was able to differentiate between participants of different UGRA experience when assessing needle tip visibility (novice 3.3 out of 10, early career 5.1, experienced anaesthetists 5.9, UGRA expert group 8.7; p < 0.01) and final needle tip placement (novice 4.2 out of 10, early career 5.6, experienced anaesthetists 6.8, UGRA expert group 8.9; p < 0.01). Subjective self-assessment by participants did not differentiate UGRA experience when assessing needle tip visibility (p = 0.07) or final needle tip placement (p = 0.07).

Discussion

An expert assessor was able to differentiate between participants with different levels of UGRA experience in this simulated environment. Objective NeedleTrainer™ and subjective participant assessments did not reach statistical significance. The findings are novel as simulated needling using live human subjects has not been assessed before, and no previous studies have attempted to objectively quantify needle tip visibility during simulated UGRA techniques. Future research should include larger sample sizes to further assess the potential use of such technology.

## Introduction

Visualising the needle is a key skill for the safe practice of ultrasound-guided regional anaesthesia (UGRA), but teaching this whilst ensuring patient safety remains a challenge [1,2]. The operator must identify the needle tip before advancing and make any subsequent required adjustments as it approaches the target [3,4]

Technology is emerging that may help track and visualise the needle tip, but it is not yet in widespread use [5,6]. There remains no agreed objective method to assess the standard of needle tip visibility during UGRA, and the authors are unaware of published data that formally quantifies operator expertise in this respect. It, therefore, remains difficult to objectively assess expertise in UGRA during training. Traditionally, technical (e.g., number of blocks preformed) and non-technical (e.g., management of the patient, recognising the limits of safe practice) factors have instead been used as surrogate markers [7,8].

A recent review article discussing future contemporary training methods in UGRA highlighted augmented reality as a key future avenue, and the Royal College of Anaesthetists UK has recently suggested the use of simulation and part-task trainers for learning in its most recent 2021 curriculum [9,10].

NeedleTrainer™ (Intelligent Ultrasound, Cardiff, UK) uses a blunt, retractable needle and augmented reality technology to superimpose a digital holographic needle on the real-time ultrasound image of a subject being scanned. This provides a simulated environment for the performance of UGRA needling skills on a real-life model. The software can also automatically calculate the amount of time the simulated needle tip is in view during any given procedure.

The device has not been validated to correlate with clinical competence or block success, so this exploratory study aims to provide data to inform future study design. However, objective knowledge of whether the needle tip is in view (using tracking technology) has been demonstrated to improve the block performance of novices [5]. Therefore, utilising simulation technologies to achieve this could be beneficial to larger groups training in regional anaesthesia.

The primary aim of this study was to determine whether there was a difference in needle tip visibility, as calculated by the device, between groups of anaesthetists with differing levels of UGRA experience.

## Materials and methods

This volunteer study was reviewed by the Office for Research Ethics Committees in Northern Ireland and deemed not to require NHS research ethics approval. The study received R&D approval via the Integrated Research Application System (IRAS; Project ID 299571).

Participants

Twenty anaesthetists from the Department of Anaesthesia, Royal Victoria Hospital, Belfast, provided written informed consent to participate in this study. Participants were stratified into four groups of five based on their experience in UGRA: novice (within the first 18 months of anaesthetic training), early career (from 18 months to 6 years into anaesthetic training), experienced anaesthetists (advanced trainees and non-expert consultants), and UGRA experts. In accordance with recent related studies, the definition of an expert was a consultant anaesthetist in the UK who met at least three of the following criteria [11-13]: (i) completed fellowship training in UGRA; (ii) holds a qualification related to UGRA (e.g., EDRA, higher degree, or equivalent); (iii) regularly delivers direct clinical care using UGRA, including for ‘awake’ surgery; (iv) regularly teaches UGRA, including regularly performing/teaching advanced techniques [14].

Models

Two volunteers provided informed written consent to be models for ultrasound scanning. The only exclusion criterion was the pathology of the areas to be scanned. The study was run over two days, with ten participants recruited each day and a different model used each day.

Expert assessor

One expert assessor (LT), meeting the criteria defined above, participated in this study. They were not blinded to the study participants.

Equipment

Ultrasound scanning was performed using a SonoSite X-Porte ultrasound machine (Fujifilm SonoSite, Bothell, Washington, USA) and a linear probe (HFL50xp). The NeedleTrainer™ device (v1.0) was connected to the X-Porte ultrasound machine via its high-definition multimedia interface output. Participants were asked to refer to the NeedleTrainer™ device screen, which displayed the ultrasound image with a superimposed virtual needle.

**Figure 1:**
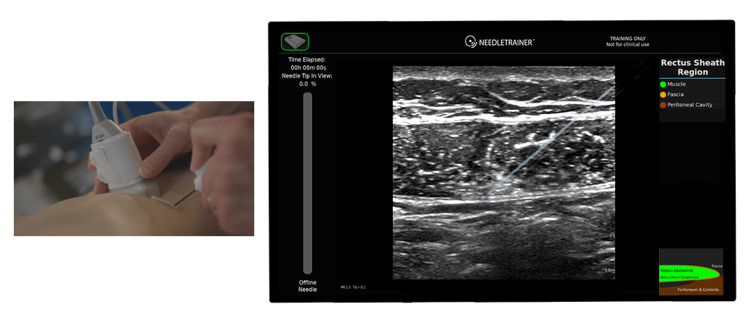
Image of a simulated rectus sheath block using the NeedleTrainer™ hardware Left image: NeedleTrainer™ transducer and needle. Right image: simulated needle on real-time ultrasound image.

NeedleTrainer™ uses electromagnetic tracking of sensors placed on the ultrasound probe, along with information from the retractable needle, to infer the relative position of the virtual needle shaft and tip, and then plots that information onscreen (Appendices, Supplementary Data A) (Figure 1).

Simulated UGRA protocol

All participants viewed a pre-recorded video prior to participation describing the three blocks to be preformed, the relevant endpoint for each block procedure, and an introduction to NeedleTrainer™. On the day of the study, hands-on familiarisation with NeedleTrainer™ was permitted prior to the assessment. This was limited to 10 minutes, with troubleshooting provided by the principal investigator (SS). Participants were permitted to practice with the NeedleTrainer™ on two block sites of the volunteer subject (the adductor canal and the forearm-level median nerve), neither of which were included in the study.

Participants then used NeedleTrainer™ to perform three simulated peripheral nerve blocks: interscalene-level brachial plexus, rectus sheath, and popliteal-level sciatic nerve [15]. These techniques involved a range of body regions (upper limb, trunk, and lower limb) and needle endpoints (perineural versus fascial plane). The target for the final simulated needle tip position was pre-defined for each block (in the earlier pre-recorded video) and represented a currently accepted standard in the literature [16,17].

Participants were free to set the ultrasound depth and gain settings deemed appropriate for each scan and use the scanning technique they would utilise in their normal clinical practice. A laminated sheet of the ideal sonographic image for all three techniques and defined NeedleTrainer™ simulated needle tip endpoints was provided to all participants. These images (Appendices, Supplementary Data B) were taken from a recent set of recommendations on structure visualisation [17], with an ‘X’ added to represent the intended final needle tip position. Assistance was permitted for initial image optimisation if required; however, there was no input once subsequent assessment with the simulated needle commenced.

The participant was timed for each procedure, from the point the retractile needle made contact with the subject’s skin to the point at which the participant declared the final needle tip position had been achieved. This timing was recorded using a start/stop function on NeedleTrainer™, which allowed the device to calculate the needle tip visibility during this period. At the end of each simulated block, the expert assessor and participant rated the attempt using the outcomes listed below. This process was repeated for all three simulated blocks.

Primary Outcome

Objective NeedleTrainer™ data: Percentage time the simulated needle tip in view, calculated by the NeedleTrainer™ software (Appendices, Supplementary Data A).

Secondary Outcomes

Expert assessor data: (i) Time taken in seconds for each simulated block attempt (recorded as per the description above). (ii) Expert assessor judgement of simulated needle tip visibility (recorded after each attempt using a 0-10 continuous visual analogue scale: 0, never visible; 10, always visible). (iii) Expert assessor judgement of accuracy of the final simulated needle tip position (0-10: 0, very poor; 10, very accurate).

Participant data: (i) Participant judgement of simulated needle tip visibility (0-10: 0, never visible; 10, always visible). (ii) Participant confidence in the final simulated needle tip position (0-10: 0, low confidence; 10, confident). (iii) Cognitive load of the entire task (participants completed a NASA task load index survey [18] following all three simulated blocks).

Sample size, data handling and analysis

As no previous studies have quantifiably assessed needle tip visibility during simulated UGRA, the authors had little data on which to base a sample size estimation. Studies using the Imperial College Surgical Assessment Device model for quantitative assessment of regional anaesthesia recruited 20-30 patients in total [19]. Therefore, a minimum of 20 participants was targeted.

Data are reported descriptively, and, where appropriate, statistical evaluation (Microsoft Excel version 2207, Build 15427.20210) was used to assess the relationship between variables. A two-factor ANOVA analysis with replication was used to compare the quantitative variables, apart from the assessment of the NASA task load index, where a single-factor ANOVA analysis was used. The Scheffe multiple comparison method was used to compare groups against each other. The null hypothesis stated that the level of experience does not influence any of the outcomes measured, with statistical significance defined as p < 0.05 (p-values are reported to 2 decimal places).

## Results

Five participants were recruited for each group (four groups, n = 20), performing a total of 60 simulated blocks. Information on participants’ experiences in UGRA can be found in Table 1 and the Appendices (Supplementary Data C).

**Table 1:** Participants self-rated experience in ultrasound-guided regional anaesthesia Each participant was asked to fill in a pre-study questionnaire regarding their experience in UGRA. They were asked to self-rate their exposure to UGRA and their needling skills on a continuous scale from 0 to 10.

	Novice group	Early career group	Experienced anaesthetist group	UGRA expert group
Self-rated UGRA exposure (0-10)	2.7	4.9	7.4	8.9
Self-rated URGA needling skills (0-10)	3.2	5.5	6.8	8.8

Both scan subjects were adult males; with body mass indexes (BMI) of 32 and 26 kg/m^2^. Primary and secondary outcomes are summarised in Table 2.

**Table 2:** Summary of data for primary and secondary outcomes Values are mean and 95% confidence intervals, with the p-value reported to 2 decimals places.

	Novice group	Early career group	Experienced anaesthetist group	UGRA expert group	P-value
Primary outcome					
% needle tip in view as per the device	41.8 (32.6–51.0)	44.5 (33.8–55.3)	43.6 (32.8–54.4)	57.1 (50.4–63.7)	0.05
Secondary outcomes					
The average time taken for a block attempt (seconds)	63.8 (42.9–84.9)	81.6 (45.4–117.8)	66.1 (33.2–98.9)	24.6 (17.0–32.2)	0.02
Expert assessor judgement of needle tip visibility (0–10)	3.3 (1.9–4.7)	5.1 (3.7–6.6)	5.9 (4.4–7.5)	8.7 (8.1–9.3)	<0.01
Expert assessor judgement of final needle tip position (0–10)	4.2 (3.1–5.3)	5.6 (4.4–6.8)	6.8 (5.5–8.1)	8.9 (8.4–9.3)	<0.01
Participant judgement of needle tip visibility (0–10)	6.1 (4.9–7.4)	6.1 (5.2–7.1)	6.5 (4.9–8.2)	7.9 (7.0–8.8)	0.07
Participant judgement of final needle tip position (0–10)	6.6 (5.7–7.4)	6.7 (5.8–7.7)	7.5 (5.9–9.2)	8.3 (7.4–9.1)	0.07

Primary Outcome

**Figure 2:**
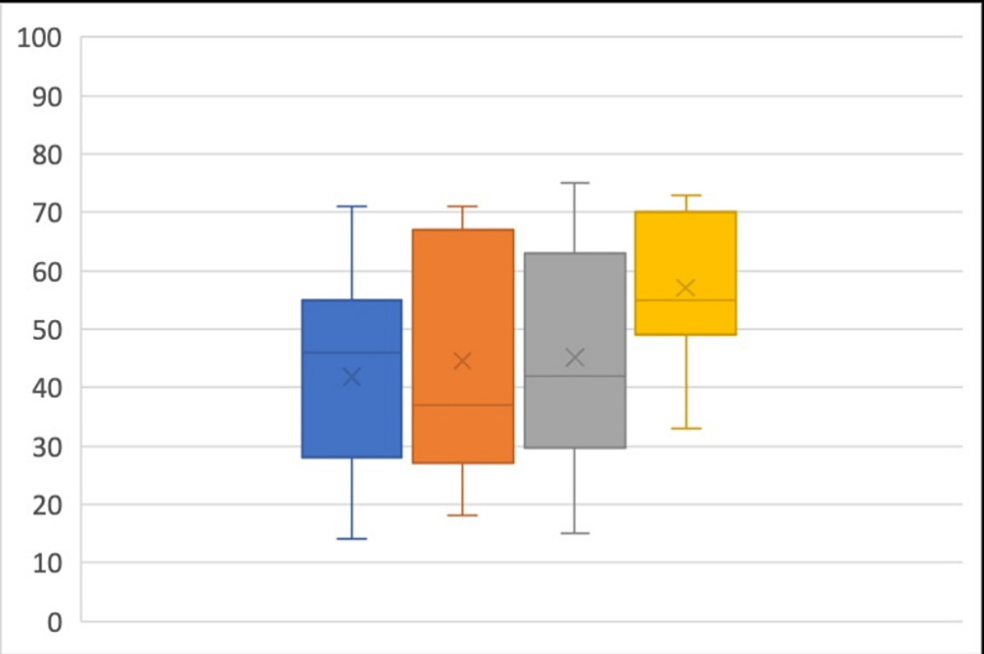
Objective NeedleTrainer™ data - percentage needle tip in view during simulated blocks Novice Group (Blue), Early Career Group (orange), Experienced Anaesthetist Group (Grey), UGRA Expert Group (yellow). Median=line; Interquartile range=box; Range=whiskers, Mean=x.

Objective NeedleTrainer™ data: Level of experience was not associated with a statistically significant difference in the percentage of time the simulated needle tip was in view (p = 0.05; Figure 2).

Secondary Outcomes

**Figure 3:**
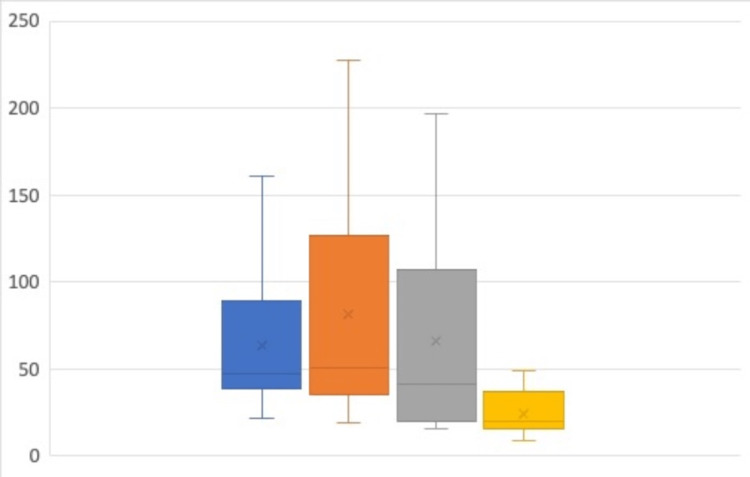
Average time (in seconds) to complete simulated regional blocks Novice Group (Blue), Early Career Group (orange), Experienced Anaesthetist Group (Grey), UGRA Expert Group (yellow). Median=line; Interquartile range=box; Range=whiskers, Mean=x.

**Figure 4:**
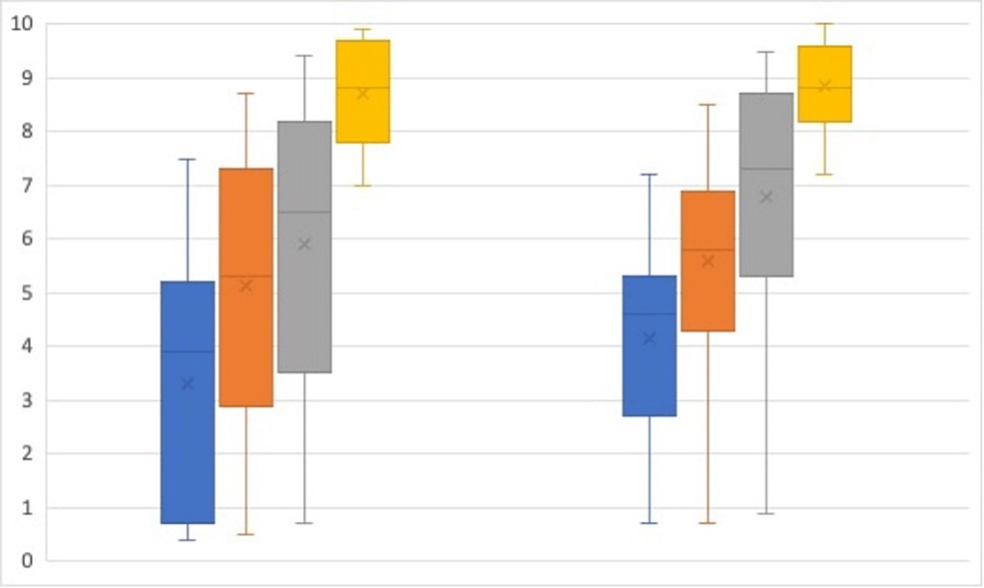
Expert assessor assessment of simulated needle tip visibility (left) and final needle tip position (right) out of 10 Novice Group (Blue), Early Career Group (orange), Experienced Anaesthetist Group (Grey), UGRA Expert Group (yellow). Median=line; Interquartile range=box; Range=whiskers, Mean=x.

Expert assessment: Level of experience was associated with a statistically significant difference in time taken to perform simulated blocks (p = 0.02; Figure 3), the expert assessor’s assessment of needle tip visibility (p < 0.01), and final needle tip placement (p-value < 0.01; Figure 4). Scheffe's analysis of the data found the UGRA expert group to be distinct from the other three groups in all three outcomes.

**Figure 5:**
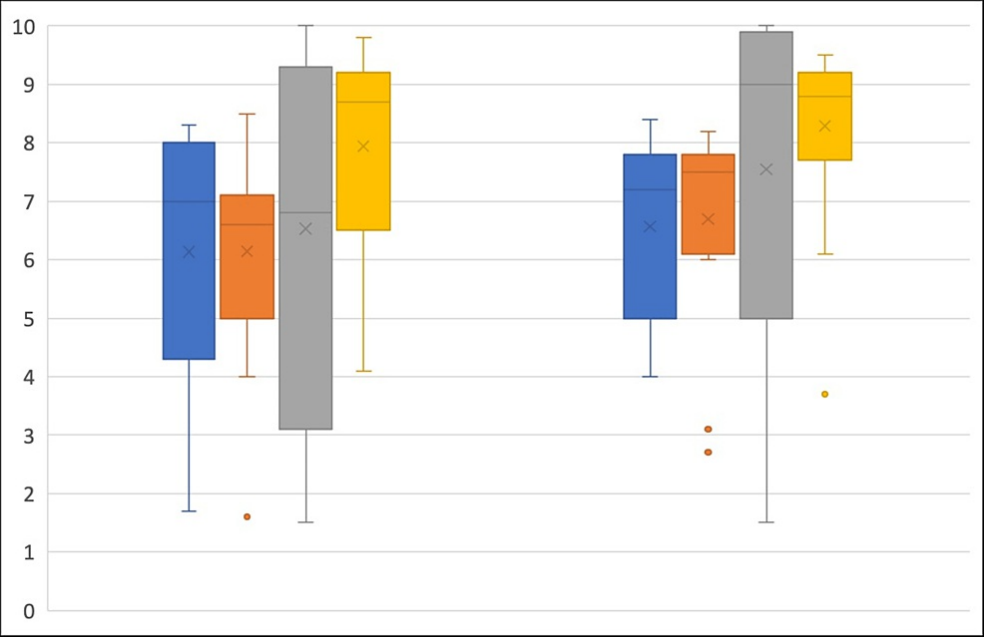
Participant assessment of needle visibility (left) and confidence in final placement of simulated needle tip (right) out of 10 Novice Group (Blue), Early Career Group (orange), Experienced Anaesthetist Group (Grey), UGRA Expert Group (yellow). Median=line; Interquartile range=box; Range=whiskers, Mean=x.

Subjective participant data: There was no difference in the participant’s own subjective assessment of needle tip visibility (p = 0.07) or confidence in the final position of the simulated needle tip (p = 0.07; Figure 5).

No variables assessed by the NASA task load index (mental demand, physical demand, temporal demand, performance, effort, and frustration) reached statistical significance when comparing stages of training (Supplementary Data D).

## Discussion

The percentage of time the needle tip was in view, as per the NeedleTrainer™ software, was higher in the expert group (95% CI 50.4-63.7%) but did not reach statistical significance (p = 0.05). The highest percentage of needle tip visibility achieved across all 60 simulated procedures, 75%, was achieved by a participant in a non-expert group, with the mean in the UGRA expert group being only 57.1%. This figure is notable as it is lower than that predicted by the authors (no previous studies have assessed this), as most UGRA experts would expect to keep their needle tip in view throughout the majority of their initial needle advancement to a target endpoint. This may represent subtleties of clinical practice not captured in this study or represent a limitation of this simulated UGRA tool, and further studies with a larger sample size are required to determine whether this is a consistent or isolated observation.

The difference in the time taken to complete simulated blocks was statistically significant, with reduced time correlating with increased experience, in keeping with the authors’ experience of clinical practice. The results support the assertion that an expert assessor is able to differentiate between different levels of operator experience, both in terms of needle visibility and simulated needle placement accuracy. The UGRA expert group took the least time and received the highest expert assessor scores. These data validate the method of human expert assessment and suggest that technology and human experts can be used in concert to assess operator performance in a simulated environment.

In comparison, the participants’ subjective assessments were not able to differentiate between experience for both needle tip visibility (p = 0.07) and final needle tip position (p = 0.07). Whilst these data may suggest the concept that anaesthetists cannot accurately determine their own level of performance, validating the current practice of external assessment, this study was not powered to examine this endpoint.

To perform UGRA independently, the user must master real-life anatomy, sono-anatomy, image acquisition/interpretation, and needle tip identification [20]. Whilst the former can be learned safely through educational events and courses, the acquisition of needle-probe orientation skills can present a significant challenge for safe development. Indeed, it has been shown in novices that at least 28 supervised attempts at the same UGRA technique are required in cadavers to achieve competence in needle visualisation [21]. Anaesthetists must develop these skills for the care of future patients whilst minimising the risk to current patients. Many low-fidelity [22] and high-fidelity [23] simulated training devices have been developed for this purpose, including hybrid and cadaveric models, with studies citing flattened learning curves, a reduction in technical errors, and fewer needle passes following their use. As well as a lack of objective assessment data, criticisms include a lack of availability and cost, particularly for high-fidelity models, which to date are not in widespread use. NeedleTrainer™ is a readily transportable device, compatible with cart-based (v1.0) and handheld (v2.0) ultrasound machines, which expands the reach of this high-fidelity augmented reality simulator to users in their own institution. It allows repeated practice of simulated UGRA on real-life ultrasound scans/subjects and can be used immediately prior to clinical practice (e.g., to remind oneself of a particular procedure or to demonstrate/practice a safe intended needle path).

The potential to collect unbiased, objective data provided by such machines may also lend itself to formal assessment if validated in future studies. This objective metric may be our current primary outcome, percentage needle tip visibility during an entire simulated block, or another metric not assessed in this study, such as the dynamic percentage needle tip in view (i.e., only whilst the simulated needle is moving).

The authors recognise limitations in this work. The small sample size (60 simulated blocks) increases the risk of a type II error, and the investigation should therefore be repeated on a larger study population. Whilst the primary outcome was objective and free from bias, the secondary outcomes were subjective. In particular, the expert assessor was unblinded when making their determinations and so may have been prone to bias if they were aware of the participant’s prior experience in UGRA. One way to prevent this in future studies is to use video recordings of each attempt, which can then be assessed by blinded expert assessors.

Participants were assessed following limited use of the device, and the study included only two scan subjects, assessing only three blocks. Therefore, future studies should include a greater range of procedures and a larger number of subjects with varying anatomical/physiological characteristics to reflect the range of clinical practice in UGRA and ensure the generalisability of these results. We also do not know if the percentage of simulated needle tip visibility necessarily correlates with clinical effectiveness, and future studies should be designed to evaluate this key outcome.

A significant challenge when designing the study protocol was categorising the four groups to be assessed, with grade of seniority used. Whilst this does not always correlate with experience/expertise in UGRA, we collected previous UGRA block numbers (Appendices, Supplementary Data C) and self-rating scores for each participant in a pre-study questionnaire. This demonstrated that in our dataset, the four groups correlated with increasing UGRA experience/exposure. Until UGRA exposure and training are standardised, it will continue to prove difficult to categorise expertise based on time in training or seniority of grade. Finally, the lack of haptic feedback through the retractile needle was noted as a potential limitation of the device compared to clinical practice, something offered by other high-fidelity simulators.

## Conclusions

In this exploratory study, we evaluated the potential utility of a novel augmented reality device, NeedleTrainer™ to differentiate between anaesthetists with varying levels of UGRA experience on real-life models. The findings are novel as simulated needling using live human subjects has not been assessed before, and no previous studies have attempted to objectively quantify needle tip visibility during simulated UGRA techniques.

The primary outcome did not reach statistical significance (p=0.05) but may have been underpowered. In this study, the combination of the NeedleTrainer™ device and an expert assessor was able to differentiate between levels of experience in UGRA. This exploratory study provides further hypothesis-generating evidence, and future studies should be focused on the validation of an objective, non-bias metric of needle visualisation that could differentiate between UGRA experiences and therefore potentially assist standardised training in the future.

## Figures and Tables

**Figure 6:**
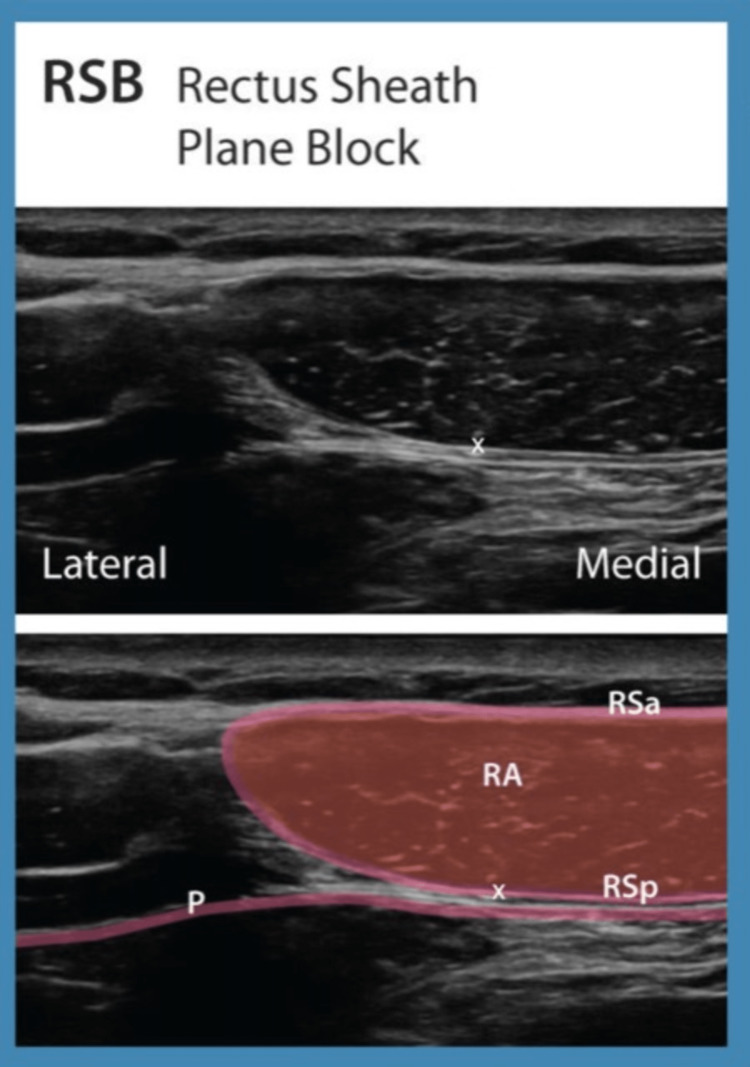
Rectus sheath block Laminated sheet provided to each participant during the online presentation and during the study session, with the target for the needle tip marked by an X.

**Figure 7:**
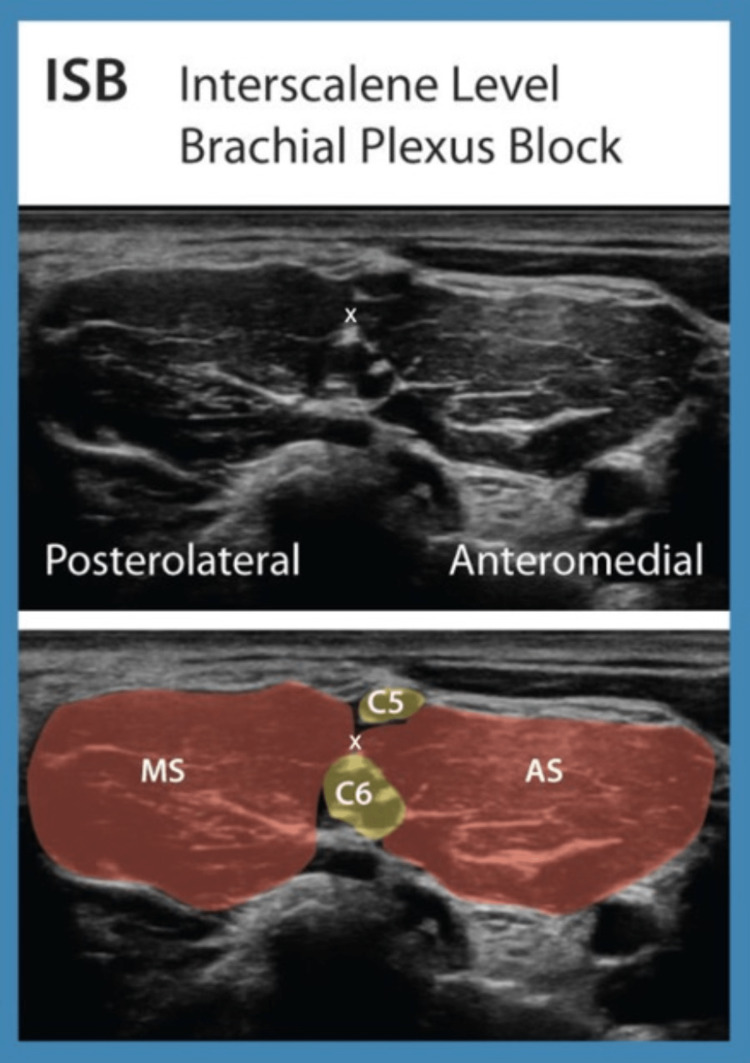
Interscalene brachial plexus block Laminated sheet provided to each participant during the online presentation and during the study session, with the target for the needle tip marked by an X.

**Figure 8:**
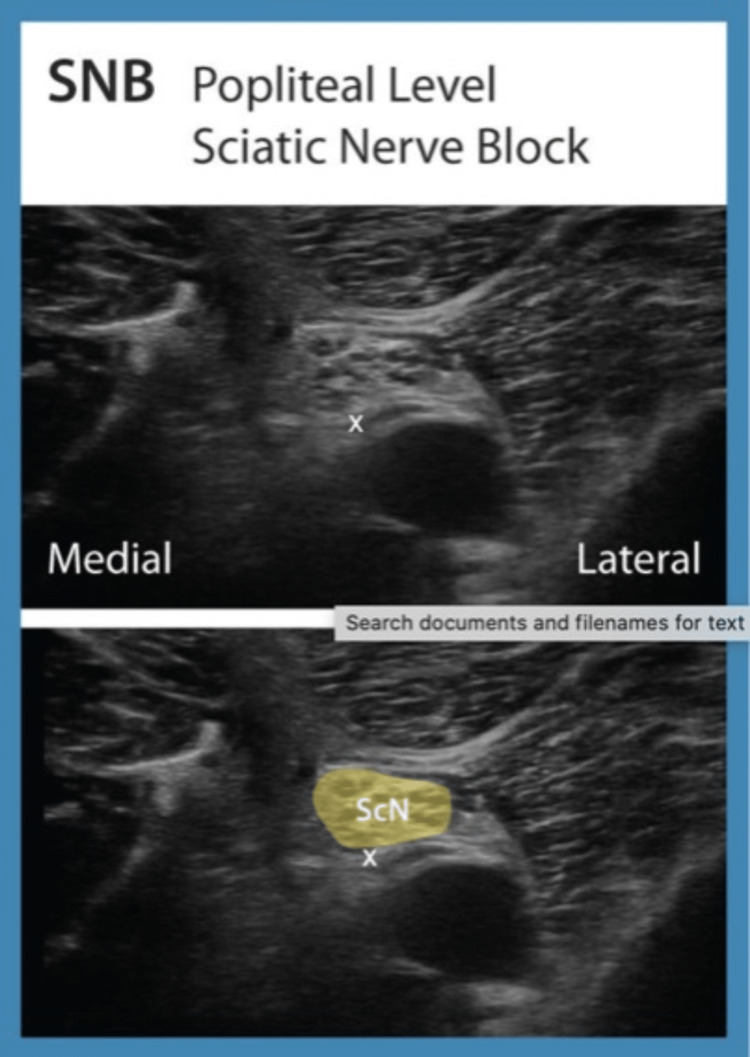
Popliteal sciatic nerve block Laminated sheet provided to each participant during the online presentation and during the study session, with the target for the needle tip marked by an X.

## Appendices

Supplementary data A

Further information on the NeedleTrainer™ System can be found below.

NeedleTrainer™ uses the Patriot electromagnetic tracking system from Polhemus [24]. This uses short-range electromagnetic fields to provide real-time tracking of the position and orientation of the sensor relative to a source transmitter (6-DOF tracking). In the NeedleTrainer™ setup, the sensor is attached to the probe, and the source is attached to the retractable needle, allowing the system to determine the relative position and orientation of the probe with respect to the retractable needle.

The ultrasound fan for the linear probe in NeedleTrainer™ is modelled as a thin virtual rectangular box with dimensions (probe width, probe depth, and ultrasound beam thickness). The NeedleTrainer™ system uses data from the tracker to calculate where the virtual needle is in relation to this box. The 'needle tip in view' metric is the proportion of time that the needle tip is inside the box, where the 'needle tip' is defined as being the last 2 mm of the needle.

The visibility of the virtual needle in this augmented reality setup is dependent on many different factors, such as the steepness of the approach (pitch axis - horizontal needles are easier to see), whether the needle is in-plane or out-of-plane (azimuth axis), the thickness of the virtual needle (low gauge needles are easier to see), the depth setting of the ultrasound machine, and the thickness of the box representing the fan (adjustable via NeedleTrainer's realism setting).

Supplementary data B

Below are pictures of the three laminated sheets provided to the participants during the online presentation and during the study session, with the target for the needle tip marked by an X (Figures 6-8).

Supplementary data C

Each participant was asked to fill out a pre-study questionnaire regarding their experience at UGRA. This included the previous overall number of blocks preformed, the previous number of each block technique to be assessed, and a self-rating of UGRA exposure/needling skills on a continuous scale of 0-10. Self-rating data are found in Table 1 in the main body. Table 3 shows the breakdown of previous UGRA blocks preformed per group.

**Table 3:** Participant's previous ultrasound-guided regional anaesthesia numbers Each participant was asked to fill in a pre-study questionnaire regarding their experience in UGRA. They were asked to estimate the number of UGRA blocks preformed to date using a modified Likert scale.

		Novice group	Early career group	Experienced anaesthetist group	UGRA expert group
Overall number of UGRA blocks	0				
	1–10	3			
	11–50	2	3		
	51–100		1		
	101–500		1	4	
	501–1000			1	2
	>1000				3
No. of rectus sheath blocks preformed	0	5			
	1–5		4	1	
	6–10		1	1	
	11–20			2	
	21–50			1	2
	>50				3
No. of interscalene blocks preformed	0	5	2		
	1–5		3	1	
	6–10				
	11–20			1	
	21–50			3	1
	>50			1	4
No. of popliteal blocks preformed	0	3			
	1–5	2			
	5–10				
	11–20				
	21–50		4	1	
	>50		1	4	5

Supplementary data D

Table 4 shows the breakdown of the NASA task load index data for each of the four groups, with corresponding p-values.

**Table 4:** Summary of average NASA task load index scores for each group

	Novice group	Early career group	Experienced anaesthetist group	UGRA expert group	P-value
Mental demand (0=very low, 20=very high)	10.0	10.4	8.8	5.4	0.28
Physical demand (0=very low, 20=very high)	2.8	7.8	3.0	2.2	0.07
Temporal demand (0=very low, 20=very high)	5.8	4.2	5.2	2.4	0.53
Performance (0=prefect, 20=failure)	9.2	8.2	5.8	5	0.49
Effort (0=very low, 20=very high)	9.6	13.6	11	7	0.25
Frustration (0=very low, 20=very high)	3.2	8.2	7.2	1.6	0.14

## References

[1] McLeod G, McKendrick M, Taylor A (2019). Validity and reliability of metrics for translation of regional anaesthesia performance from cadavers to patients. Br J Anaesth.

[2] Ahmed OM, O'Donnell BD, Gallagher AG, Shorten GD (2017). Development of performance and error metrics for ultrasound-guided axillary brachial plexus block. Adv Med Educ Pract.

[3] Abdallah FW, Macfarlane AJ, Brull R (2016). The requisites of needle-to-nerve proximity for ultrasound-guided regional anesthesia: a scoping review of the evidence. Reg Anesth Pain Med.

[4] Woodworth G, Maniker RB, Spofford CM (2020). Anesthesia residency training in regional anesthesiology and acute pain medicine: a competency-based model curriculum. Reg Anesth Pain Med.

[5] McLeod GA, McKendrick M, Taylor A (2020). An initial evaluation of the effect of a novel regional block needle with tip-tracking technology on the novice performance of cadaveric ultrasound-guided sciatic nerve block. Anaesthesia.

[6] Tielens LK, Damen RB, Lerou JG, Scheffer GJ, Bruhn J (2014). Ultrasound-guided needle handling using a guidance positioning system in a phantom. Anaesthesia.

[7] Smith AF, Pope C, Goodwin D, Mort M (2006). What defines expertise in regional anaesthesia? An observational analysis of practice. Br J Anaesth.

[8] Carraccio CL, Benson BJ, Nixon LJ, Derstine PL (2008). From the educational bench to the clinical bedside: translating the Dreyfus developmental model to the learning of clinical skills. Acad Med.

[9] Ramlogan RR, Chuan A, Mariano ER (2021). Contemporary training methods in regional anaesthesia: fundamentals and innovations. Anaesthesia.

[10] (2022). 2021 Anaesthetics Curriculum, RCOA. Anaesthetics.

[11] Bowness JS, El-Boghdadly K, Woodworth G, Noble JA, Higham H, Burckett-St Laurent D (2022). Exploring the utility of assistive artificial intelligence for ultrasound scanning in regional anesthesia. Reg Anesth Pain Med.

[12] Bowness JS, Burckett-St Laurent D, Hernandez N (2023). Assistive artificial intelligence for ultrasound image interpretation in regional anaesthesia: an external validation study. Br J Anaesth.

[13] Bowness JS, Macfarlane AJ, Burckett-St Laurent D (2023). Evaluation of the impact of assistive artificial intelligence on ultrasound scanning for regional anaesthesia. Br J Anaesth.

[14] Ashken T, Bowness J, Macfarlane AJ (2022). Recommendations for anatomical structures to identify on ultrasound for the performance of intermediate and advanced blocks in ultrasound-guided regional anesthesia. Reg Anesth Pain Med.

[15] Turbitt LR, Mariano ER, El-Boghdadly K (2020). Future directions in regional anaesthesia: not just for the cognoscenti. Anaesthesia.

[16] El-Boghdadly K, Wolmarans M, Stengel AD (2021). Standardizing nomenclature in regional anesthesia: an ASRA-ESRA Delphi consensus study of abdominal wall, paraspinal, and chest wall blocks. Reg Anesth Pain Med.

[17] Bowness JS, Pawa A, Turbitt L (2022). International consensus on anatomical structures to identify on ultrasound for the performance of basic blocks in ultrasound-guided regional anesthesia. Reg Anesth Pain Med.

[18] Sandra G, Hart LE (1988). Development of NASA-TLX (task load index): results of empirical and theoretical research. Adv Psychol.

[19] Chin KJ, Tse C, Chan V, Tan JS, Lupu CM, Hayter M (2011). Hand motion analysis using the imperial college surgical assessment device: validation of a novel and objective performance measure in ultrasound-guided peripheral nerve blockade. Reg Anesth Pain Med.

[20] Bowness J, El-Boghdadly K, Burckett-St Laurent D (2021). Artificial intelligence for image interpretation in ultrasound-guided regional anaesthesia. Anaesthesia.

[21] Barrington MJ, Wong DM, Slater B, Ivanusic JJ, Ovens M (2012). Ultrasound-guided regional anesthesia: how much practice do novices require before achieving competency in ultrasound needle visualization using a cadaver model. Reg Anesth Pain Med.

[22] Liu Y, Glass NL, Glover CD, Power RW, Watcha MF (2013). Comparison of the development of performance skills in ultrasound-guided regional anesthesia simulations with different phantom models. Simul Healthc.

[23] McLeod G, Eisma R, Schwab A, Corner G, Soames R, Cochran S (2010). An evaluation of Thiel-embalmed cadavers for ultrasound-based regional anaesthesia training and research. Ultrasound.

[24] Chmarra MK, Grimbergen CA, Dankelman J (2007). Systems for tracking minimally invasive surgical instruments. Minim Invasive Ther Allied Technol.

